# Time point-independent tumor positivity of ^68^Ga-PSMA-PET/CT pre- and post-biopsy in high-risk prostate cancer

**DOI:** 10.1007/s12149-022-01732-w

**Published:** 2022-04-01

**Authors:** Sijuan Zou, Shuang Song, Jianyuan Zhou, Bo Yu, Dong Kuang, Zhihua Wang, Xiaohua Zhu

**Affiliations:** 1 Department of Nuclear Medicine, Tongji Hospital, Tongji Medical College, Huazhong University of Science and Technology, 1095 Jiefang Ave, Wuhan, 430030 Hubei China; 2 Department of Pathology, Tongji Hospital, Tongji Medical College, Huazhong University of Science and Technology, Wuhan, China; 3 Department of Urology, Tongji Hospital, Tongji Medical College, Huazhong University of Science and Technology, Wuhan, China

**Keywords:** Positron emission tomography (PET)/computed tomography (CT), Prostate cancer, Prostate-specific membrane antigen (PSMA), Prostate biopsy, High risk

## Abstract

**Objective:**

Prostate-specific membrane antigen (PSMA)-PET/CT imaging has gained increasing clinical importance for the detection and staging of high-risk primary prostate cancer (PCa). However, it is unclear whether the routine practice of prostate biopsy obscures the image finding of PSMA-PET/CT. This study aimed to compare the tumor positivity rate of PSMA-PET/CT performed pre- (PSMA-PET/CT_pre_) and post-biopsy (PSMA-PET/CT_post_) in high-risk PCa patients.

**Patients and methods:**

We matched 58 PSMA-PET/CT_post_ with 58 PSMA-PET/CT_pre_ studies for primary detection of high-risk PCa according to clinical characteristics. Three subgroups of PSMA-PET/CT_post_ were defined by the intervals after biopsy (≤ 1 week, 1 ~ 2 weeks, and 2 ~ 5 weeks). Tumor positivity rates were determined, and SUVmax of primary tumors were compared separately for the two main groups and the related subgroups. Malignant prostate tissues from 20 of these patients were examined by immunohistochemical analysis of PSMA. In addition, the values of PSMA-PET/CT_pre_ and PSMA-PET/CT_post_ in assessing seminal vesicle invasion (SVI) were evaluated in patients who underwent radical prostatectomy.

**Results:**

All the primary tumors were positive on PSMA-PET/CT_post_ and PSMA-PET/CT_pre_ imaging, resulting in a patient-based positivity rates of 100% (58/58) in both groups. All examined IHC results (20/20) confirmed the high-level expression of PSMA. SUVmax of primary tumors did not differ between the two main groups (16.1, IQR 9.8–26.6 vs. 16.5, IQR 11.0–26.7, *p* > 0.05). Subgroup analysis of PSMA-PET/CT_post_ (≤ 1 week, 1 ~ 2 weeks, and 2 ~ 5 weeks) also showed no significant difference in tumor SUVmax (15.8, IQR 9.5–22.2; 17.8, IQR 9.8–29.2; and 15.4, IQR 10.1–30.3. *p* > 0.05). PSMA-PET/CT_post_ and PSMA-PET/CT_pre_ exhibited similar value in SVI detection as well.

**Conclusions:**

The tumor positivity rate was consistently high for PSMA-PET/CT pre- and post-biopsy. A prior biopsy does not seem to affect the tumor positivity rate of PSMA-PET/CT in high-risk PCa.

**Supplementary Information:**

The online version contains supplementary material available at 10.1007/s12149-022-01732-w.

## Introduction

Prostate cancer (PCa) is currently the most common neoplasm in males and the second leading cause of cancer-related deaths for males in western countries [1]. Standard procedure for PCa diagnosis includes detection of elevated prostate-specific antigen (PSA) level and digital rectal examination (DRE) in patients [2].

Imaging techniques opens up a new horizontal for the diagnosis and management of males linked with risk of prostate cancer. Multiparametric magnetic resonance imaging (mpMRI), for example, proves to be a valuable tool for risk stratification, biopsy guidance, and local staging of clinically significant PCa [3, 4]. The procedure of invasive prostate biopsy, however, may cause biopsy-related artifacts including hemorrhage and edema in the imaging results, and subsequently lead to over- or under-estimation of tumor burden. As a result, prostate MRI is usually performed prior to a biopsy and the use of MRI in PCa management within a short time after biopsy should be cautioned [5, 6], especially in those with previous negative or inconclusive biopsy reports.

Prostate-specific membrane antigen (PSMA) is a transmembrane protein with a 100- to 1000-times higher expression in primary and metastatic prostate tumors than in normal tissues [7, 8]. PSMA-targeted positron emission tomography (PET) is increasingly used to assess the recurrence of PCa as well as to localize primary disease and metastases [9–17]. The recent prospective PRIMARY trial [18] showed that the combination of PSMA-PET and mpMRI was superior to mpMRI alone in the diagnosis of clinically significant PCa. While PSMA-PET gains popularity as the primary diagnostic modality for PCa, it is also brought to attention that a pre-imaging biopsy may compromise the performance of PSMA-PET/CT or PSMA-PET/MR, in a similar way to that of MRI. On the other hand, the imaging results of PSMA-PET/CT are reconstructed from the distribution of PSMA molecules rather than anatomical features, and therefore, may be unaffected by the biopsy-related artifacts. Up to date, it is unclear if a biopsy affects the tumor positivity rate of the ensuing PSMA-PET/CT for primary PCa, or whether it is necessary to implicate a waiting-period between the biopsy and the following PSMA-PET/CT.

Therefore, we herein present this retrospective, matched-pair analysis to assess the tumor positivity rate of pre- and post-biopsy ^68^Ga-PSMA-PET/CT in patients with high-risk PCa.

## Patients and methods

### Patients

Fifty-eight patients (mean age 68.1 ± 7.8 years; range 50–81 years) with newly diagnosed high-risk PCa who underwent post-biopsy ^68^Ga-PSMA-617 PET/CT (PSMA-PET/CT_post_) at our institution from April 2018 to December 2020 were consecutively enrolled and retrospectively analyzed. Based on the European Association Urology guidelines, high-risk patients were defined as those with the presence of one or more of the following criteria: PSA concentration > 20 ng/mL, ISUP grade group ≥ 4, or clinical stage ≥ T2c [19]. Of these, 48 had undergone a transrectal ultrasonography (TRUS)-guided transrectal 12-core prostate biopsy, 1 had trans-perineal mpMRI targeted biopsy (8 + 1-core) and 9 outpatient received biopsy in other institutions with unknown approaches and number of needle cores. All patients were examined by ^68^Ga-PSMA-PET/CT within 5 weeks after prostate biopsy (PSMA-PET/CT_post_) and were subcategorized into three subgroups based on the interval between biopsy and PSMA-PET/CT: ≤ 1 week, 1 ~ 2 weeks, and 2 ~ 5 weeks. Patients with a history of electron-prostatectomy or being treated with anti-cancer therapy prior to PSMA-PET/CT scan were excluded. PSA values tested within 5 weeks before PSMA-PET/CT were identified. Available post-biopsy MRI data of these patients for pretreatment local staging were also reviewed to determine the presence or absence of hemorrhage. Of the 58 PSMA-PET/CT_post_ patients, 28 underwent radical prostatectomy (RP) within 1 month after the PSMA-PET/CT scan.

Fifty-eight corresponding patients (mean age 69.7 ± 8.7 years; range 46–85 years) with pathology-confirmed PCa who had undergone ^68^Ga-PSMA-PET/CT before prostate biopsy (PSMA-PET/CT_pre_) during the same time period were consecutively identified in the institution’s database on the basis of the following clinical parameters: ISUP grade group (2–3 vs. 4–5), pre-scan PSA values (4–20 ng/mL, > 20 ≤ 100 ng/mL vs. > 100 ng/mL and clinical Tumor stage (cT) (cT2c, cT3 vs. cT4). Characteristics of the matched-pair cohorts are summarized in Table 1. For PSMA-PET/CT_pre_ patients, there were longer time intervals between biopsy, radical prostatectomy, and the post-biopsy PSMA-PET, which resulted in patient dropouts from the study. Of the 58 PSMA-PET/CT_pre_ patients, 14 underwent RP within 1 month after the examination.

**Table 1:** Patient characteristics

Characteristics		PSMA-PET/CT_post_	PSMA-PET/CT_pre_
No		58	58
Age, years, mean ± SD		68.1 ± 7.8	69.7 ± 8.7
ISUP grade	2–3 4–5	11 47	11 47
tPSA (ng/mL) median (IQR)	4–20 > 20 ≤ 100 > 100	76.5 (34.5–183.5) 3 34 21	77.6 (38.6–187.2) 3 34 21
cT (clinical T stage)	cT2 cT3 cT4	4 42 12	4 41 13

This study was approved by the Ethics Committee of the Tongji Hospital, Tongji Medical College, Huazhong University of Science and Technology (No. TJ-IRB20190422). All reported investigations were conducted in accordance with the Helsinki Declaration and national regulations. Patient data were de-identified and processed per institutional ethics guidelines.

### Imaging procedure

^68^Ga-PSMA-617 was administered as an intravenous bolus at a dose of 89–200 MBq (mean 114.6 ± 30.9 MBq) and PET acquisition was started at a mean ± SD time of 60 ± 11 min after tracer injection on a dedicated PET/CT hybrid tomography (GE Discovery 690, General Electronic Healthcare, USA). Image acquisition was started with a non-enhanced helical CT scan using automatic mA-modulation and 120 kV. CT scans were reconstructed to a slice thickness of 3.75 mm. PET scans were acquired in 3D mode with an acquisition time of 120 ~ 180 s per bed position from the mid-thighs to the skull base. PET images were reconstructed with the built-in GE VUE point method.

### PET image analysis and quantification

All PSMA-PET/CT_post_ and PSMA-PET/CT_pre_ imaging were evaluated by two board-certified nuclear medicine physicians with specific training in ^68^Ga-PSMA-PET/CT in consensus. Readers were blinded to patients’ history and histopathological results. The scan was considered positive when intraprostatic tracer uptake higher than liver was noticed as reported in the PROstate cancer Molecular Imaging Standardized Evaluation (PROMISE) study [20]. Tumor uptake patterns were classified as unilateral focal, bilateral multifocal, and increased diffuse uptake. Maximum standardized uptake value (SUVmax) of the suspected prostate tumors with the highest PSMA-ligand uptake was noted. Seminal vesicle invasion was defined by the presence of focal or diffuse PSMA-ligand accumulation above the background by visual analysis. PROMISE criteria were adopted for interpretation of PSMA uptake in lymph node and bone metastases [20]. Tumor positivity rates were determined separately for PSMA-PET/CT_post_ and PSMA-PET/CT_pre_ group. SUV_max_ of primary tumors were compared in (1) the PSMA-PET/CT_post_ and PSMA-PET/CT_pre_ groups, (2) three subgroups of PSMA-PET/CT_post_ patients with different intervals after biopsy (≤ 1 week, 1 ~ 2 weeks and 2 ~ 5 weeks), (3) patients with organ-confined tumor and those with locally advanced PCa (pT2 vs. ≥ pT3) and (4) patients with and without PSMA-avid lymph nodes (N) and bone metastases (BM): (N+ BM+, N− BM+, N+ BM− and N− BM−). The correlations of tumor SUVmax with the tPSA level, ISUP grade, and pT were also analyzed.

### Pathological and immunohistochemical (IHC) analyses

A total of 42 (28 from PSMA-PET/CT_post_ group and 14 from PSMA-PET/CT_pre_ group) patients underwent radical prostatectomy with resection of the seminal vesicles. PSMA was stained with an anti-PSMA rabbit monoclonal antibody (EPR6253, ab133579, Abcam, 1:500 dilution) on a Leica Bond-Max auto-stainer. The intensity of staining (weak, moderate or intense) and the percentage of positively stained cells (focal, regional, or diffuse) were graded as reported in a previous study [21]. Cases categorized as intense diffuse, intense regional, or moderate diffuse were considered as overexpressing PSMA protein.

### Statistical analysis

Statistical analyses were performed using SPSS version 26.0 software (IBM Corp., Armonk, New York). Quantitative data are expressed as mean values ± standard deviations (SD) or medium ± interquartile range (IQR). Tumor positivity rates were determined separately for the two groups. The sensitivity, specificity and accuracy of PSMA-PET/CT for evaluating SVI were calculated using histopathology results of RP as the reference standard. For comparisons of the tumor SUVmax between groups and in subgroups, *p* values were calculated by the Mann–Whitney *U* test. The correlations of tumor SUVmax with the tPSA level, ISUP grade, and pT were analyzed using Spearman’s correlation. *p* values < 0.05 were considered statistically significant.

## Results

### Tumor positivity rate of ^68^Ga-PSMA-PET/CT_post_ and ^68^Ga-PSMA-PET/CT_pre_

#### Primary tumor and PSMA protein expression by IHC

All the primary tumors were positive on PSMA-PET/CT_post_ and PSMA-PET/CT_pre_ (both 100%, 58/58) imaging. Twenty prostatectomy specimens from these primary tumors were available for immunohistochemistry, all of which showed strong expression of PSMA.

The SUVmax values of primary tumors did not differ between the PSMA-PET/CT_post_ and PSMA-PET/CT_pre_ groups (16.1, IQR 9.8–26.6 vs. 16.5, IQR 11.0–26.7, *p* > 0.05). Neither did the subgroup analysis of PSMA-PET/CT_post_ (≤ 1 week, 1 ~ 2 weeks, and 2 ~ 5 weeks) show any significant difference in the SUVmax of tumor (15.8, IQR 9.5–22.2; 17.8, IQR 9.8–29.2; and 15.4, IQR 10.1–30.3. *p* > 0.05) (Table 2). In terms of the pattern of tumor uptake, PSMA-PET/CT revealed diffuse infiltration, bilateral focal and unilateral focal prostate tumor in 64% (37/58), 17% (10/58) and 19% (11/58) of PSMA-PET/CT_post_ patients, respectively, and in 64% (37/58), 15% (9/58) and 21% (12/58) of PSMA-PET/CT_pre_ patients in comparison (Supplemental Fig. 1).

**Table 2:** Comparison of SUV_max_ of the primary tumor

Patients	No. of patients	SUVmax median (IQR)	*p* value
PSMA-PET/CT_post_ group	58	16.1 (9.8–26.6)	*p* = 0.669
PSMA-PET/CT_pre_ group	58	16.5 (11.0–26.7)	
Subgroups of PSMA-PET/CT_post_ interval after biopsy ≤ 1 week > 1 ≤ 2 weeks > 2 ≤ 5 weeks	(*n* = 58) 34 18 6	15.8 (9.5–22.2) 17.8 (9.8–29.2) 15.4 (10.1–30.3)	*p* = 0.842
RP patients pT pT2 ≥ pT3	(*n* = 42) 7 (16.7%) 35 (83.3%)	9.5 (7.9–16.8) 16.3 (11.4–30.0)	*p = 0.041
All patients N+ BM+ N− BM+ N+ BM− N− BM−	(*n* = 116) 49 (42.3%) 13 (11.2%) 20 (17.2%) 34 (29.3%)	16.6 (9.8–25.8) 11.5 (9.7–17.9) 24.4 (15.2–33.4) 15.9 (10.4–19.2)	*p = 0.043

*RP* radical prostatectomy, *N* lymph nodes, *BM* bone metastasis

*p < 0.05

**Fig. 1:**
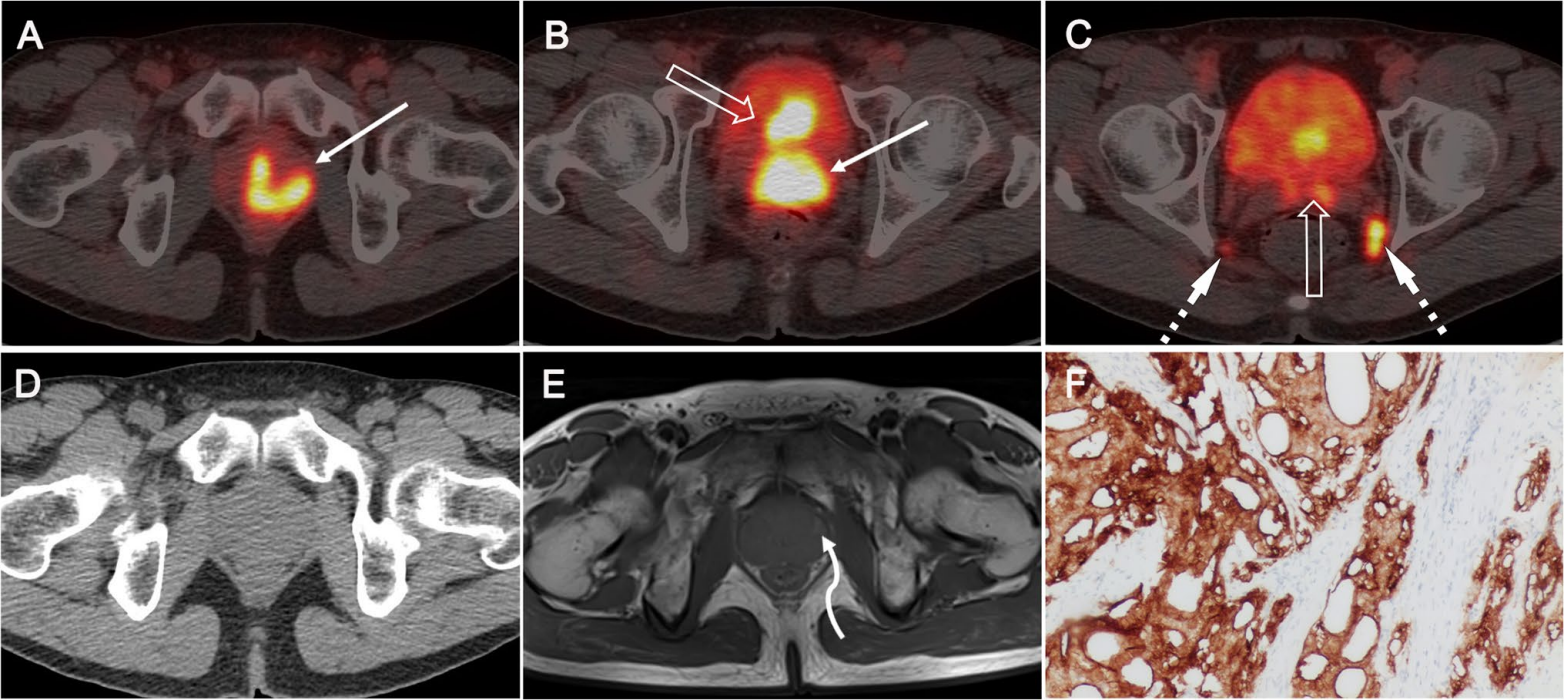
Large primary tumors 5 days after prostate biopsy on ^68^Ga-PSMA-PET/CT. A 56-year-old patient with newly diagnosed PCa (tPSA 95 ng/mL, ISUP 5), underwent pretreatment ^68^Ga-PSMA-PET/CT 5 days after biopsy (**A–D**), transaxial fused images show diffuse tracer accumulation in the primary (SUVmax 31.1, solid arrows in **A** and **B**) and extension of the tumor to the bladder (open arrow in **B**) with SVI on both sides (open arrow in **C**) and metastases to pelvic lymph nodes (dot arrows in **C**). Corresponding axial CT image present without typical malignant findings (**D**). Post-biopsy MR image for pretreatment local staging were obtained 3 days after the biopsy. Prostate lesions were assigned a Prostate Imaging Reporting and Data System (PI-RADS) score of 5. T1-weighted image (**E**) shows minor hemorrhage (curve arrow) as the high-signal-intensity area in the left peripheral zone. Immunohistochemical staining showing that the primary tumors were highly positive for PSMA (**F**)

Moreover, the SUVmax of primary tumor was significantly higher in patients with locally advanced PCa (≥ pT3; 16.3, IQR 11.4–30.0) as compared to that in organ-confined PCa (pT2; 9.5, IQR 7.9–16.8) (*p* = 0.041). Difference in the uptake by primary tumor was also recorded among individuals with and without metastases: N+ BM+ (42.3%, 49/116, median 16.6), N− BM+ (11.2%, 13/116, 11.5), N+ BM− (17.2%, 20/116, 24.4) and N− BM− (29.3%, 34/116, 15.9), respectively, *p* = 0.043) (Table 2 and Supplemental Fig. 2).

**Fig. 2:**
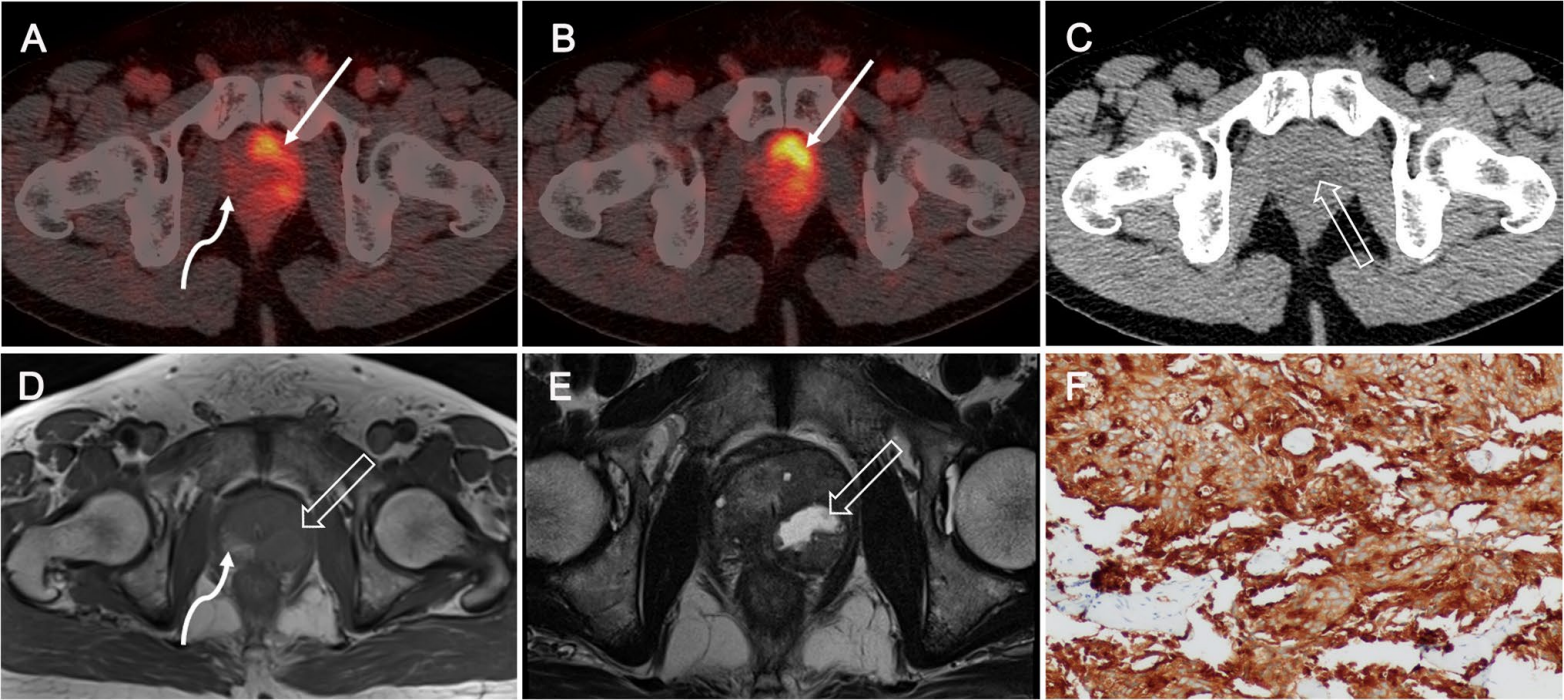
Primary tumors 1 week after prostate biopsy on ^68^Ga-PSMA-PET/CT. A 69-year-old patient with biopsy-proved PCa (tPSA 48 ng/mL, ISUP 5). Pre-operative ^68^Ga-PSMA-PET/CT performed 1 week after biopsy (**A**–**C**), transaxial fused images show irregular increased radiotracer uptake (SUVmax 12.1, solid arrows in **A** and **B**) in both prostate lobes. Corresponding CT image shows an equivocal low-density (open arrow in **C**) in the prostate. Post-biopsy MR images (**D**–**E**) for local staging obtained 5 days after biopsy show the prostate tumor, qualified as PI-RADS category 5, with central necrosis in the left lobe as isointense signal intensity on T1-weighted MR image and heterogeneous (isointense to high) signal intensity on T2-weighted MR image (open arrows in **D** and **E**). In addition, T1-weighted MR image shows hemorrhage (**D**, curve arrow) as a high-signal-intensity area in the right peripheral zone, with no increased tracer uptake on the corresponding PET/CT image (curve arrow in **A**). Immunohistochemistry of tumor section after RP showing strong PSMA staining in the primary tumors (**F**)

**Fig. 3:**
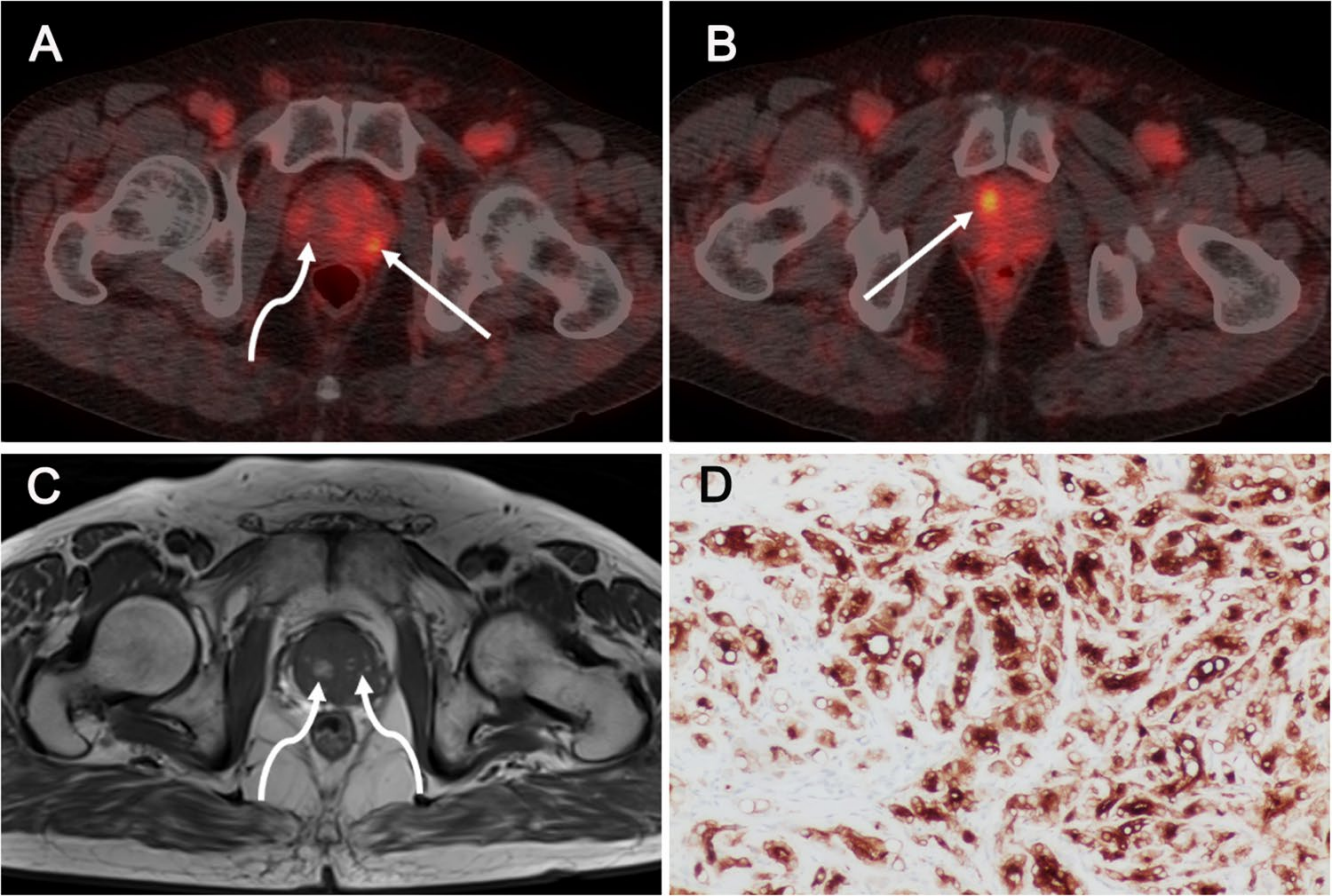
Bilateral small primary tumors 3 weeks after biopsy on ^68^Ga-PSMA-PET/CT. A 74-year-old man with PCa referred to our hospital. He had undergone biopsy in an external center (ISUP 5, tPSA 16 ng/mL) 3 weeks ago. ^68^Ga-PSMA-PET/CT_post_ images (**A**–**B**) show bilateral focal positive lesions in the prostate (SUVmax 5.7 and 7.9, solid arrows in **A** and **B**). Post-biopsy MR image obtained 2 days before PSMA-PET reveled lesions qualify as PI-RADS score 3 and 4. T1-weighted MR (**C**) image shows multiple hemorrhage as the high-signal-intensity area in the prostate (curve arrows in **C**), with no increased tracer uptake on corresponding PET/CT image (curve arrow in **A**). PSMA-positive immunohistochemical staining of primary tumors after RP (**D**)

**Fig. 4:**
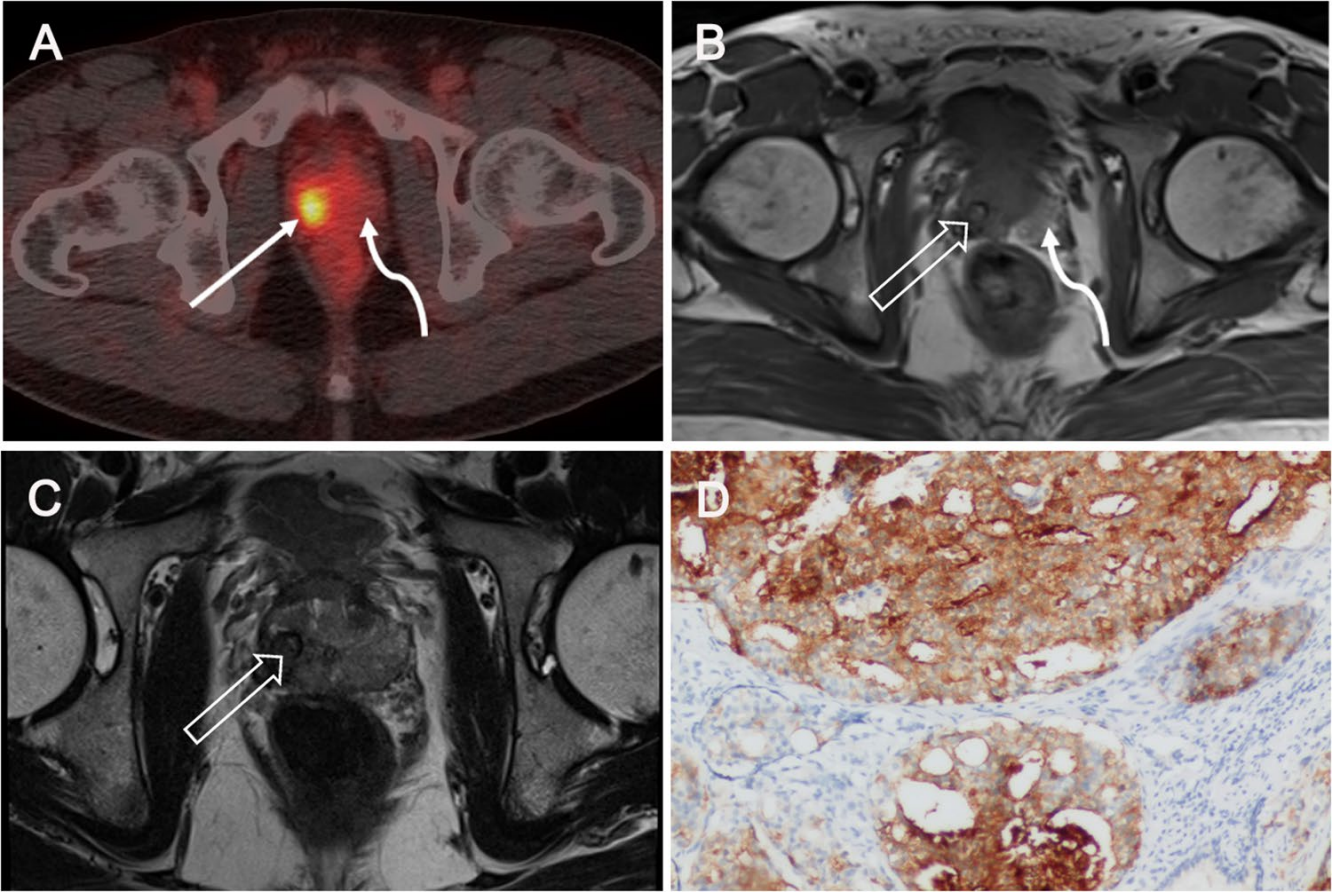
Unilateral small primary tumor 1 month after biopsy on ^68^Ga-PSMA-PET/CT. The 54-year-old man was admitted to our hospital after biopsy in an external center (ISUP 4, tPSA 25 ng/mL) a month ago. ^68^Ga-PSMA-PET/CT_post_ image (**A**) shows a focal positive lesion (SUVmax 10.3, solid arrow in **A**) in the right lobe of the prostate. Post-biopsy MR images (**B**–**C**) obtained 2 days before PSMA-PET, both T1 and T2-weighted MR image show a prostate lesion in the right peripheral zone with a clear dark rim indicating past hemorrhage after biopsy (open arrows in **B** and **C**). In addition, T1-weighted MR image shows high signal intensity in the left peripheral zone indicating post-biopsy hemorrhage (curve arrow in **B**), with no increased tracer uptake on the corresponding PET/CT image (curve arrow in **A**). Immunohistochemical staining for PSMA showing strong expression in the primary tumors after RP (**D**)

**Fig. 5:**
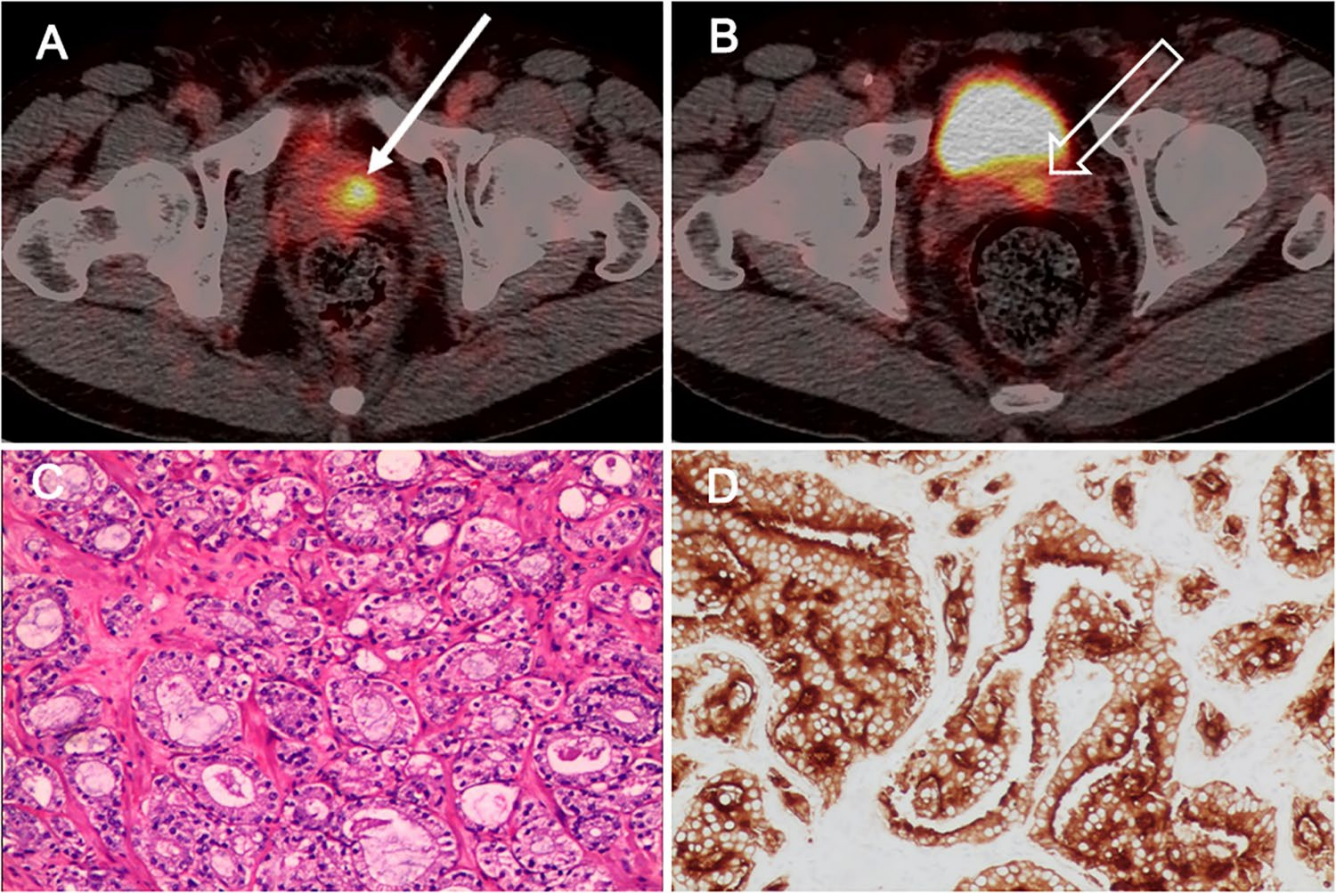
Increased tumor uptake of ^68^Ga-PSMA in the prostate prior to biopsy. A 68-year-old man with a tPSA level of 24 ng/mL. ^68^Ga-PSMA-PET/CT_pre_ images (**A**) show positive lesion (SUVmax 11.2, solid arrow in **A**) in the left lobe of the prostate and focal tracer uptake in the left seminal vesicle (open arrow in **B**). The pathological results (**C**) after RP demonstrate PCa (ISUP 2) with tumor invasion of the left seminal vesicle. Immunohistochemical tests present a remarkable PSMA expression in the primary tumors (**D**)

Post-biopsy MR imaging for preoperative local staging was available for review in eight of the PSMA-PET/CT_post_ patients, and hemorrhages was observed in seven of them. In contrast to the intense PSMA-ligand uptake in prostate tumors, the uptake in hemorrhage regions was minimal. Representative images of ^68^Ga-PSMA-617 scans are shown in Figs. 1–5: primary tumor at 5 days (Fig. 1) and 1 week (Fig. 2) after prostate biopsy, bilateral focal lesions in prostate at 3 weeks after biopsy (Fig. 3), single focal lesion in prostate at 1 month after biopsy (Fig. 4), and increased tumor uptake prior to biopsy (Fig. 5).

### Seminal vesicle invasion (SVI)

Using histopathological results of resected samples as the reference standard, the sensitivity, specificity and accuracy of imaged-based SVI detection were calculated as the following: PSMA-PET/CT_post_: 84.6% (11/13), 100.0% (15/15), and 92.9% (26/28); PSMA-PET/CT_pre_: 87.5% (7/8), 100.0% (6/6), and 92.9% (13/14) (Table 3). Therefore, pre- and post-biopsy PSMA-PET/CT scans exhibited similar diagnostic performance in SVI detection.

**Table 3:** Accuracy of ^68^Ga-PSMA-PET/CT for detection of histopathologically proven invasion of seminal vesicles in patients with high-risk prostate cancer

	Sensitivity	Specificity	Accuracy
^68^Ga-PSMA-PET/CT_post_ (*n* = 28)	84.6% (11/13)	100% (15/15)	92.9% (26/28)
^68^Ga-PSMA-PET/CT_pre_ (*n* = 14)	87.5% (7/8)	100% (6/6)	92.9% (13/14)

### Correlation of tumor SUVmax in PSMA-PET/CT and other parameters

**Fig. 6:**
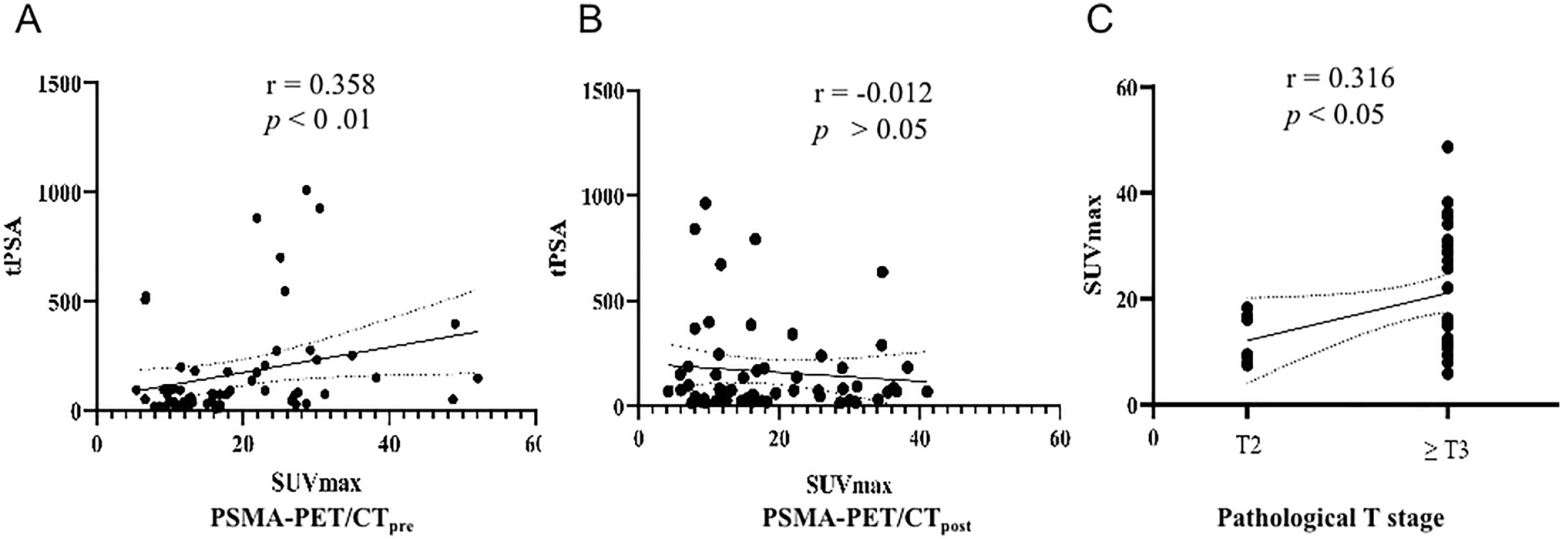
**A** The tumor SUV_max_ was positively correlated with the tPSA value in PSMA-PET/CT_pre_ patients. **B** No significant correlation between tumor SUV_max_ and pre-scan tPSA value was found in the PSMA-PET/CT_post_ patients. **C** A slightly positive correlation was also found between tumor uptake of PSMA and the pT stage in patients who underwent RP

A slightly positive correlation was observed between tumor SUVmax and the pT stage (*r* = 0.316, *p* < 0.05) in patients who underwent RP. There was a similar positive correlation between the tumor SUVmax and tPSA value (*r* = 0.358, *p* < 0.01) in PSMA-PET/CT_pre_ patients. However, no significant correlation between tumor SUVmax and pre-scan tPSA value was found in the PSMA-PET/CT_post_ patients (*r* = − 0.012, *p* > 0.05) (Fig. 6). Neither was a correlation established between ISUP grade and the tumor SUVmax (*r* = 0.085, *p* > 0.05).

## Discussion

With the current shift towards an early PSMA-PET/CT scan for the primary diagnosis and staging of PCa, it is imperative to understand the potential impact of biopsy on the imaging quality and diagnostic power of the ensuing PET. Previous studies have demonstrated that PSMA-PET/CT could yield remarkable results for the pre-surgery identification of primary high-risk PCa and metastases [22–24]. For example, Fendler et al. [25] reported that ^68^Ga-PSMA-PET/CT performed minimally 1-week post-biopsy could present satisfactory diagnostic accuracy for the detection of primary lesions. Nevertheless, the time interval between biopsy and the following PET/CT has yet to be specified in most of the studies. Therefore, we postulated that an evaluation on the impact of biopsy timing upon the tumor positivity rate of PSMA-PET/CT would help fill such a knowledge gap.

Our results demonstrate the pre- and post-biopsy PSMA-PET/CT both detected 100% of primary high-risk PCa lesions, showing no significant difference in SUV max of the primary tumors. Subgroup analysis of patients undergoing PSMA-PET/CT_post_ revealed that the time interval between biopsy and ensuing PET scan did not have a significant impact on the SUVmax values of tumors. Neither did this time interval have any effects on the performance of PET/CT in locating tumor nodules. Taken together, we have shown that biopsy is unlikely to compromise the diagnostic power of ensuing PSMA-PET/CT for primary or metastatic lesions, and any delay of the post-biopsy PET scan is unnecessary. Our results provide valuable guidelines for clinicians, as an example, in scheduling a timely PSMA-PET scan for a high-risk patient that has a negative or inconclusive biopsy report.

Hemorrhage is the most frequent complication in prostate biopsy, and has been detected in MRI scans on 72.2%, 57.1%, and 52% patients at < 4, 4 ~ 6, and > 6 weeks after the biopsy, respectively [26]. While there have been no reports on the impact of biopsy-induced hemorrhage on the detectability of PSMA-PET in PCa, our study confirmed that hemorrhage or blood products did not take up radiotracers in PSMA-PET. In comparison, there was an intense uptake of radiotracers in pathologically verified prostate tumors. Therefore, we concluded that in spite of biopsy-induced hemorrhage, PSMA-PET/CT was capable of detecting primary tumor lesions in patients with high-risk PCa, regardless of the time interval between biopsy and PET.

PSAM-PET/CT’s ability to withstand the interference by a prior biopsy can be attributed to the following factors. First, the high PSMA expression in PCa enables a specific imaging of PSMA molecules in prostate tumors [7, 8]. Preclinical studies have shown that prostate tumors exhibited highly homogenous and intense expression of PSMA [27, 28]. The IHC results of patients in our study also validated that PSMA was homogenously overexpressed throughout the prostate tumors, which constituted the molecular pre-requisite for increased tumor uptake of PSMA ligands. Second, the blood products of hemorrhage did not take up PSMA ligands. Third, the extent of post-biopsy hemorrhage was less severe in prostate tumor than in peritumor normal tissues, which became even less significant as the tumor size increased and the percentage of normal tissues dropped [29]. Notably, 64% of patients with advanced and large tumors in both study groups were presented with diffuse intense tumor uptake on PSMA-PET imaging, enabling PSAM-PET/CT ability to offset the interference. Fourth, the biopsy-related hemorrhage in tumor foci may have resolved spontaneously more rapidly than in normal prostatic tissue [30]. Lastly, PSMA expression in inflammatory lesions was reported to be extremely rare [7].

In our results, the tumor positivity rates and SVI detection value for ^68^Ga-PSMA-PET/CT_post_ and ^68^Ga-PSMA-PET/CT_pre_ are slightly higher than those reported in the previous studies [23, 29, 31–33], probably because PSA screening for early detection of PCa has not been widely adopted and therefore more patients already had advanced-stage tumors at the time of PET scans [34].

In line with previous studies, the tumor SUVmax was found to be higher in locally advanced PCa (≥ pT3) than organ-confined tumor (pT2) (*p* < 0.05) in patients who underwent RP (Table 2). However, the radiotracer uptake of primary lesions in patients with metastatic PCa was not consistently higher than that in patients without metastasis. Mannweiler et al. reported that metastatic PCa exhibited significant intra- and inter- tumor heterogeneity [35]. Silver et al. showed that metastatic lesions tended to have higher PSMA expression than primary lesions, whereas nodule metastases had higher PSMA expression than bone metastases [7]. Therefore, the variability of the intraprostatic uptake of PSMA-ligand may be caused by the inherent heterogeneity of metastatic PCa. The intensity of intraprostatic tracer uptake, however, did not have any impact on patient management in these metastatic cases.

Moreover, a slightly positive correlation was found between tumor uptake of PSMA and the pT stage in patients who underwent RP as well as the tumor SUVmax and tPSA value in PSMA-PET/CT_pre_ patients. These findings were consistent with those of previous studies [22, 24, 36]. However, no significant correlation between the tumor SUVmax and pre-scan tPSA value was found in the PSMA-PET/CT_post_ patients. This could be attributed to the spurious transient elevation of serum PSA associated with mechanical manipulation of the prostate by biopsy or catheterization in the post-biopsy cohort [37]. In addition, we did not observe a significant correlation between the tumor SUVmax and the ISUP grade, which can be explained in part by the small number of patients with ISUP grade 2 and 3 and the potential disagreement of ISUP grade between biopsy and prostatectomy specimens might be the underlying reasons [38].

Our study has some limitations. First, this is a retrospective matched-pair comparison in high-risk PCa patients conducted in a single institute. Second, the limited sample size and distribution of cancer risk population may also cause a potential bias. Third, the needle biopsy of the prostate was performed by physicians from different institutions, and their technique or the number of needle cores varied. In addition, a direct comparison between PSMA-PET/CT_post_ with post-biopsy MRI imaging was not available for the whole cohort, as MRI is not mandatory in the post-biopsy setting in our institution. Meanwhile, whole-mount step-section of pathologic specimens from prostatectomy was not performed as a routine analysis. Thus, PSMA-PET/CT for PCa diagnosis was not evaluated using lesion-based analysis. Therefore, further investigations using a head-to-head comparison of PSMA-PET performed before and after prostate biopsy in a greater spectrum of cancer risk population are warranted.

## Conclusion

The tumor positivity rate was consistently high for PSMA-PET/CT pre- and post-biopsy. A prior biopsy does not seem to affect the tumor positivity rate of PSMA-PET/CT in high-risk PCa.

## Supplementary Information

Below is the link to the electronic supplementary material.

Supplementary file 1 (TIF 2025 KB) Tumor uptake pattern of the prostate on PSMA PET/CT_post_and PSMA PET/CT_pre_image.

Supplementary file 2 (TIF 2025 KB) Differences in primary tumor uptake were recorded between surgery patients with different pT stage (**A**) as well as in patients with and without metastatic disease (**B**, N+ BM+, N-BM+, N+ BM-and N-BM-).
