# Supplementary figures and images for: Time point-independent tumor positivity of 68Ga-PSMA-PET/CT pre- and post-biopsy in high-risk prostate cancer
Source: Ann Nucl Med. 2022 Apr 1;36(6):523–32. doi: 10.1007/s12149-022-01732-w (PMC9132805; doi:10.1007/s12149-022-01732-w)

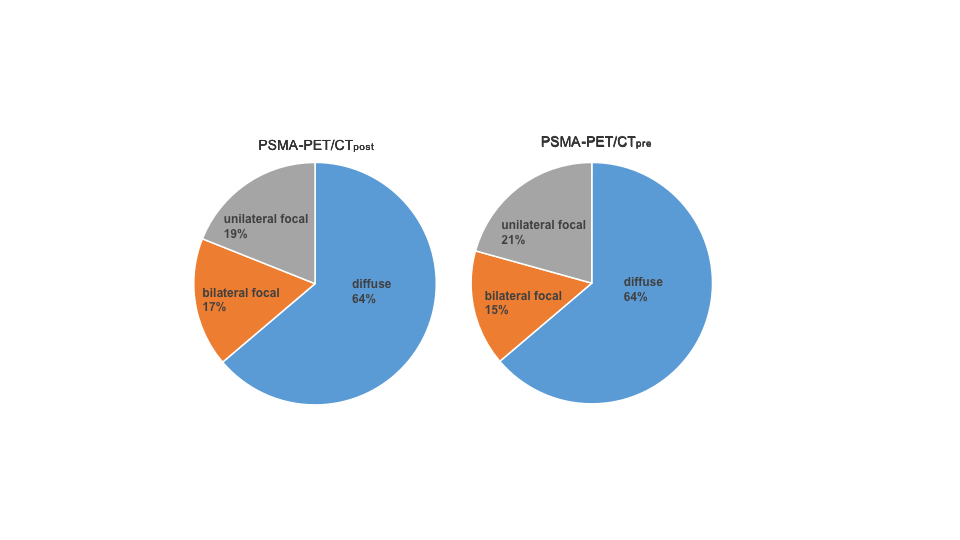

Supplement: Supplementary file 1 — (TIF 2025 KB) Tumor uptake pattern of the prostate on PSMA PET/CTpostand PSMA PET/CTpreimage. [file 12149_2022_1732_MOESM1_ESM.tif]

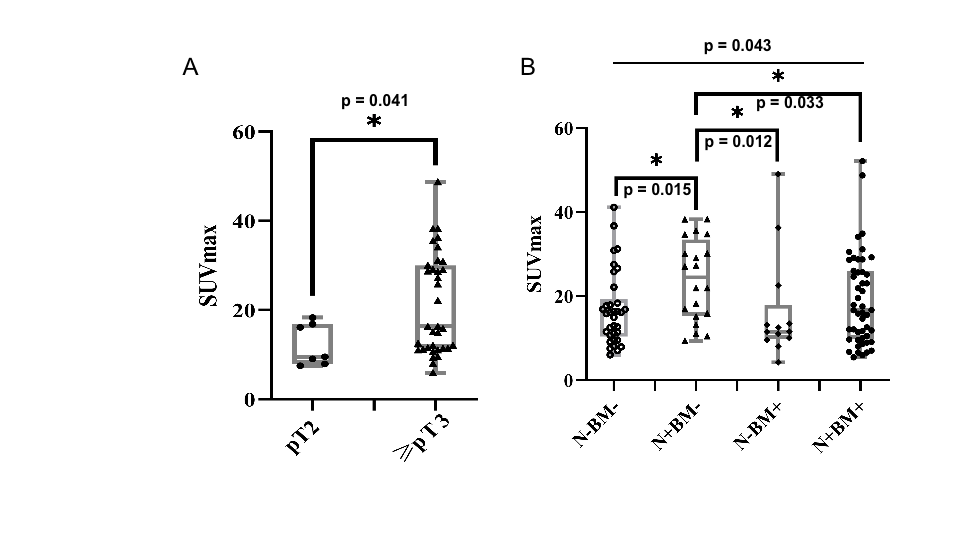

Supplement: Supplementary file 2 — (TIF 2025 KB) Differences in primary tumor uptake were recorded between surgery patients with different pT stage (A) as well as in patients with and without metastatic disease (B, N+ BM+, N-BM+, N+ BM-and N-BM-). [file 12149_2022_1732_MOESM2_ESM.tif]
